# Supplementary material for: Methodology for predicting hospital admissions and evaluating recovery rates for coronavirus disease in Japan
Source: PLoS One. 2025 Oct 23;20(10):e0334643. doi: 10.1371/journal.pone.0334643 (PMC12548901; doi:10.1371/journal.pone.0334643)
Supplement: Attachment — Submitted filename: Response to reviewers.docx [file pone.0334643.s002.docx]

**Response to reviewers**

Thank you for giving me the opportunity to work on your response letter. I have checked each of the responses for tone, language, clarity, and correspondence with the reviewers’ comments and the changes made in the revised manuscript. I wish you the best for the resubmission of your revised manuscript.

Reviewer #1: The manuscript presents a methodology for predicting hospital admissions for COVID-19

in Japan by integrating a simple SIR model with observed relationships between new positive cases and

hospitalization data. The work also extends the approach to estimate recovery rates, enabling

retrospective evaluation of medical interventions and policy measures. The topic is timely and relevant, and the dataset used is comprehensive, covering the entire Japanese population. The manuscript is

generally well-structured and clearly written, with a logical flow from problem statement to

methodology, results, and implications but needs to address the below issues.

1. While the model performs well with Japanese national data, it is unclear how adaptable the

methodology is to regions with different healthcare systems, testing policies, or hospitalization criteria.

A brief discussion of applicability to other contexts would improve the manuscript’s impact.

Thank you for your positive feedback and constructive comments. As stated on line 295, viz. “The recovery rate could be determined for each hospital, and the results would be useful in initiating supportive measures for the hospital.”, the relationship between the number of new inpatients and number of hospitalized patients treated at each hospital corresponds to P and H, respectively, in this study. Therefore, the same prediction method should be applicable to hospitals in any country. However, due to the small absolute numbers, the fluctuations caused by various factors in the data were not offset, leading to reduced accuracy in the prediction of the number of hospitalizations and determination of the recovery rate. Aggregating this data at the prefectural or national level allowed averaging the data of over millions of people, making it possible to categorically determine the intrinsic recovery rate of the variant strain.

The following sentences were inserted into the manuscript:

“Equation (1) is a “conservation equation” for the number of hospitalized patients. This relationship should hold true for any closed system, irrespective of the complexity of the model employed.”

“However, applying this to each hospital is expected to reveal differences in factors specific to each hospital, such as disease severity, age, and comorbidities of the patients, and the healthcare system. If the data collection system utilized in this study can be established as a countermeasure in the next pandemic, it could be widely adopted internationally to determine recovery rates in mathematical models and predict bed shortages.”

2. The approach assumes that all new positive cases requiring treatment are tracked in the dataset. As

acknowledged in the limitations, this assumption no longer holds in many contexts. It would be useful to

explore adjustments or correction factors for partial case tracking.

Thank you for your insightful comments. The limitations described in this study pertain to the data collection methods at the national level. Individual hospitals should be able to manage inpatient numbers internally; therefore, while the findings of this study may contain increased error due to the reduction in the total numbers, they can be applied without restriction to clinical practice predictions.

3. The model’s predictive performance is reported primarily in terms of concordance rates. However,

there is no formal uncertainty analysis (e.g., confidence intervals, sensitivity analysis) to assess

robustness under varying data quality or parameter uncertainty.

Thank you for your insightful comments.

The following sentence was inserted into the manuscript:

“The coefficient of determination between the two was R² = 0.993. The test period was 985 days, from January 16, 2020, when the data became available, to September 26, 2022.”

4. While the recovery rate (γ) is used effectively for retrospective evaluation, the causal

interpretation—particularly its linkage to vaccination campaigns—relies on temporal coincidence.

Additional statistical testing or correlation analysis would strengthen these claims.

Thank you for your insightful comments. We identified the possibility of the vaccination campaign as a potential causal factor of recovery based on temporal coincidence. However, no other nationwide campaigns or temporary nationwide events showing the same temporal coincidence were found in government-published articles at the national level during this investigation. We agree that additional statistical testing is necessary to definitively determine a causal relationship with vaccination.

The following sentence was inserted into the manuscript:

“Additional statistical verification is required before establishing definitive causality between the recovery rate and vaccination.”

Reviewer #2: The manuscript addresses a public health challenge, i.e. predicting hospital admissions and recovery rates during the COVID-19 pandemic in Japan. The approach combines a simple SIR model with empirical hospitalization data. The findings, particularly the ability to estimate recovery rates and hospitalization peaks, are relevant for pandemic preparedness and healthcare resource planning. However, the manuscript requires major revisions.

1. The main claimed novelty is the integration of hospitalization data to refine recovery rates. The manuscript should more clearly articulate what distinguishes this work from existing SIQR or SEIR modeling studies.

Thank you for your constructive comments. Input parameters are treated as variables in the SIQR and SEIR models, akin to the SIR model. Their values are swept across a range for exploration, or values representing formulated hypotheses are used. When used as constants, values reported in clinical research data for recovery are employed. Recovery rate values from clinical research are often based on patient progress at several hospitals and, while estimated, are likely based on a scale of approximately 100 patients. This study represents the average value for a population of 10 million people. Moreover, it refines the recovery rate as a time-varying parameter, which constitutes its novelty.

The following description was inserted into the manuscript:

“Equation (1) is a “conservation equation” for the number of hospitalized patients. This relationship should hold true for any closed system, irrespective of the complexity of the model employed.” “However, applying this to each hospital is expected to reveal differences in factors specific to each hospital, such as disease severity, age, and comorbidities of the patients, and the healthcare system.”

2. The discussion should elaborate on how this methodology could be generalized beyond COVID-19 or Japan, which would strengthen its broader applicability.

Thank you for your comments. The limitations described in this study pertain to the data collection methods at the national level. Individual hospitals should be able to manage inpatient numbers internally; therefore, while the findings of this study may contain increased error due to the reduction in the total numbers, they can be applied without restriction to clinical practice predictions.

3. The prediction accuracy of “99% concordance” is mentioned several times. This figure is very high and requires a clear explanation of how it was calculated, i.e. R², mean absolute percentage error or concordance correlation coefficient.

Thank you for your insightful comments. Thank you for your insightful comments.

Accordingly, the following sentence was inserted into the manuscript:

“The coefficient of determination between the two was R² = 0.993. The test period was 985 days, from January 16, 2020, when the data became available, to September 26, 2022.”

4. The paper should provide sensitivity analyses showing how results change with different assumptions about γ.

Thank you for your comments. The γ values for each prefecture in this study represent the average recovery rate for millions of people. Due to the enormity of this scale, further calculations under different assumptions are impractical. This study focused on discussing the overall spread of the epidemic and did not entail detailed investigation of γ.

5. The validation is based solely on retrospective Japanese data. To strengthen the case for the model, the author should attempt an out-of-sample validation, i.e. training on data from one wave, testing on another or using one prefecture as training and another for validation.

Thank you for your comments. To reach the conclusions about this study's methodology, data from Japan's prefectures is sufficient. While the need for out-of-sample validation is understandable, its purpose is to confirm whether different values of γ can be obtained; it does not alter the conclusions regarding the methodology itself. Since we do not expect the same γ value, dividing the data into training and validation sets is meaningless. Applying this method to different regions or time periods represents an attempt to expand its applicability for exploring new factors (such as the properties of new variants or changes in immunity among the Japanese population).

6. Comparison with more complex models, i.e. machine learning based predictions or SEIR models, would help demonstrate advantages and trade-offs of this simpler approach.

Thank you for your comments. Equation (1) is essentially a hospital patient number conservation equation. This relationship should hold true regardless of the complexity of the model employed. This simple approach is the most straightforward way to arrive at the essence. In contrast, machine learning requires feeding in vast amounts of big data, and complex models also necessitate preparing big data due to the involvement of many uncertain parameters.

If we were to compare it to more complex models, we would need to prepare many different types of big data to determine these complex models, which would deviate from the purpose of this research.

7. The manuscript equates recovery rates derived from hospitalization data with those of community acquired infection models. This assumption might not be fully justified since hospitalized patients may differ systematically from the broader infected population. The author should discuss this.

Thank you for your comments. Certainly, the recovery rate derived from the hospitalization data cannot be considered equivalent to the recovery rate in the community infection model. However, the recovery rate γ for community infections is unknown because measured values are unavailable. Furthermore, the data on the number of infected individuals that should be examined for consistency is the number of new positive cases. The fact that the number of new positive cases is well reproduced using a reliable γ value for hospitalized patients suggests that the recovery rate for community infection can also be considered to be of a similar magnitude. This means that any researcher using a mathematical model should apply a promising γ value using the same rationale.

The corresponding text in the manuscript was rewritten as follows.

“Studies using the equivalent SIR model of the chain reaction [15,19], which closely reproduces the Tokyo data, have reported an anomaly, i.e., a sudden reduction in the infection rate during the same two periods as $\gamma_{larger-ex}$. Reflecting the increased recovery rate of $\gamma_{larger-ex}$ during these periods resolved this anomaly in the new positive case data proportional to community-acquired infections.”

8. The attribution of higher recovery rates to vaccination campaigns is plausible but speculative. The discussion should be more cautious and reference direct evidence linking vaccination timing to observed recovery rates.

Thank you for your insightful comments. We identified the possibility of the vaccination campaign as a potential causal factor of recovery based on temporal coincidence. However, no other nationwide campaigns or temporary nationwide events showing the same temporal coincidence were found in government-published articles at the national level during this investigation. We agree that additional statistical testing is necessary to definitively determine a causal relationship with vaccination.

The following sentence was inserted into the manuscript:

“Additional statistical verification is required before establishing definitive causality between the recovery rate and vaccination.”

9. While some limitations are acknowledged others are underdeveloped. Additional limitations that should be explicitly discussed include: Exclusion of age, comorbidities and regional healthcare capacity; reliance on reported “positives” which may not reflect true infection rates due to testing capacity and policy changes; assumption that all positive cases initially require treatment / quarantine which is no longer accurate.

Thank you for your comments. As noted in the Discussion section, a limitation of this method is that not all new positive cases are treated and quarantined. When using the number of new admissions P at a given hospital as the input rather than new positive cases, the recovery rate for that hospital should be determined over time. Furthermore, this should enable prediction of hospital overcrowding based on the number of inpatients H. The recovery rate in this case would be determined using multivariate analysis considering various factors such as disease severity, age, and comorbidities of the hospitalized patients, treatment systems, and testing capacity. This would clarify differences in recovery rates among hospitals and the underlying reasons.

This can be confirmed by the distribution of γ across all prefectures shown in Figure 4, revealing regional differences in recovery rates. Specifically, significant variations in γ values across prefectures are observed during each peak of the infection spread. This is likely attributable to the above-mentioned factors not pursued in this study. Detailed investigation into these factors requires specific inquiries at each hospital. Unless such data are publicly available, issues of ethics approval and patient consent arise, making it difficult to readily use such data for analysis. Crucially, the focus of this study was on proposing a prediction method, not on investigating the factors underlying the recovery rates.

The following text was inserted into the manuscript:

“Equation (1) is a “conservation equation” for the number of hospitalized patients. This relationship should hold true for any closed system, irrespective of the complexity of the model employed.”

“However, applying this to each hospital is expected to reveal differences in factors specific to each hospital, such as disease severity, age, and comorbidities of the patients, and the healthcare system. If the data collection system utilized in this study can be established as a countermeasure in the next pandemic, it could be widely adopted internationally to determine recovery rates in mathematical models and predict bed shortages.”

10. Some references are heavily Japan centric. Including more international studies on hospitalization prediction would situate the work broadly in the literature.

Thank you for your comments. Japan-centric data proved sufficient for proposing the predictive methodology advocated in this study. Moving forward, as a countermeasure in the next pandemic, it would be desirable to adopt the same data acquisition method used for Japan’s 990-day COVID-19 pandemic period. Preparatory research must be conducted internationally. This will enable widespread application the determination of recovery rates and prediction of bed shortages using mathematical models internationally.

The following sentences were inserted into the manuscript:

“If the data collection system utilized in this study can be established as a countermeasure in the next pandemic, it could be widely adopted internationally to determine recovery rates in mathematical models and predict bed shortages.”
